# Association between *H. pylori* infection status, UBT-derived DOB level, and colorectal polyp detection

**DOI:** 10.3389/fonc.2026.1851795

**Published:** 2026-06-09

**Authors:** Yu Zhou, Ge Yu, Zhigang Huang, Rong Wan

**Affiliations:** 1 Department of Gastroenterology, Xuancheng People’s Hospital, Affiliated Xuancheng Hospital Wannan Medical University, Xuancheng, Anhui, China; 2 ShanghaiQ General Hospital of Nanjing Medical University, Shanghai, China; 3 Department of Gastroenterology, Shanghai General Hospital, Shanghai Jiaotong University School of Medicine, Shanghai, China

**Keywords:** ^13^C-urea breath test, adenomatous polyps, colorectal polyps, delta over baseline, *Helicobacter pylori*

## Abstract

**Objective:**

To investigate the associations of Helicobacter pylori (*H. pylori*) infection status and urea breath test-derived delta over baseline (DOB) values with colorectal polyp detection, and DOB-related associations among *H. pylori*-positive participants.

**Methods:**

This single-center ambispective observational study included 439 adults who underwent both a ¹³C-urea breath test (¹³C-UBT) and total colonoscopy during the same diagnostic workup at our hospital between January 2023 and December 2024. *H. pylori* positivity was defined as a DOB value ≥4.0‰. The primary outcome was colorectal polyp detection, and adenomatous polyps were analyzed as a secondary outcome. Logistic regression models were used in the unmatched cohort, and generalized estimating equation models were applied after one-to-one propensity score matching. Additional analyses were performed among *H. pylori*-positive participants using ln(DOB) and DOB quartiles. Exploratory receiver operating characteristic analysis was performed in the unmatched cohort.

**Results:**

In the unmatched cohort, the detection rate of colorectal polyps was higher in the *H. pylori*-positive group than in the *H. pylori*-negative group. After propensity score matching, 144 matched pairs were obtained, and the detection rate remained higher in the *H. pylori*-positive group (68.75% *vs* 52.78%). In the unmatched cohort, *H. pylori* positivity was associated with higher odds of colorectal polyp detection after multivariable adjustment (OR = 4.05, 95% CI: 2.60–6.30, P<0.001). In the matched cohort, this association persisted in generalized estimating equation models (adjusted OR = 1.91, 95% CI: 1.19–3.07, P = 0.005). Higher DOB levels were associated with colorectal polyp detection in unmatched and matched analyses. Among *H. pylori*-positive participants, ln(DOB) and higher DOB quartiles were associated with increased odds of colorectal polyp detection. Adenomatous polyps were more frequently detected in the *H. pylori*-positive group (50.00% *vs* 35.42%, P = 0.017). Exploratory ROC analysis showed an AUC of 0.713.

**Conclusion:**

In this single-center ambispective cohort, *H. pylori* positivity and higher urea breath test-derived DOB values were associated with increased odds of colorectal polyp detection. Similar DOB-related associations were observed among *H. pylori*-positive participants, and adenomatous polyps were more frequently detected in the positive group. However, DOB alone showed limited discriminatory ability, and these findings require validation in larger multicenter studies.

## 1 Introduction

Colorectal cancer is one of the most common malignant tumors worldwide and is associated with high incidence and mortality rates (1). In recent years, the overall incidence of colorectal cancer in China has continued to rise, and in some regions, a trend toward younger age at onset has also been observed (2). Colorectal cancer usually develops through a multistep process from normal mucosa to adenomatous polyps and eventually to invasive cancer (3). Colorectal polyps are an important precancerous condition associated with colorectal cancer; early detection and treatment can reduce the risk of developing colorectal cancer (4). In addition to traditional risk factors, identifying novel and readily accessible markers for risk stratification may help optimize the allocation of limited colonoscopy resources and improve screening efficiency.

*Helicobacter pylori* (*H. pylori*) infection remains common in China (5, 6). In addition to its established associations with gastritis and peptic ulcer disease, the possible extragastric effects of *H. pylori*, especially its relationship with colorectal mucosal lesions, have attracted increasing attention in recent years (7). A number of cross-sectional studies, case-control studies, cohort studies, as well as systematic reviews and meta-analyses have investigated the association between *H. pylori* infection and colorectal lesions from different perspectives (8). Most observational studies suggest that individuals positive for *H. pylori* have a higher risk of colorectal polyps or adenomas than those who are negative, and some studies have further reported associations between *H. pylori* infection and an increased risk of multiple polyps, large polyps, and advanced adenomas (9). However, the existing evidence remains inconsistent (10, 11).

The results of the ¹³C-UBT are usually expressed as DOB. Higher DOB values indicate stronger *H. pylori* urease activity in the stomach and, to some extent, reflect current infection activity (12, 13). However, few studies have explored the association between the degree of infection activity and the risk of colorectal lesions. Therefore, the present study aimed to investigate this association not only from the perspective of infection status, but also from the perspective of UBT-derived DOB level.

## 2 Materials and methods

### 2.1 Study population

**Figure 1:**
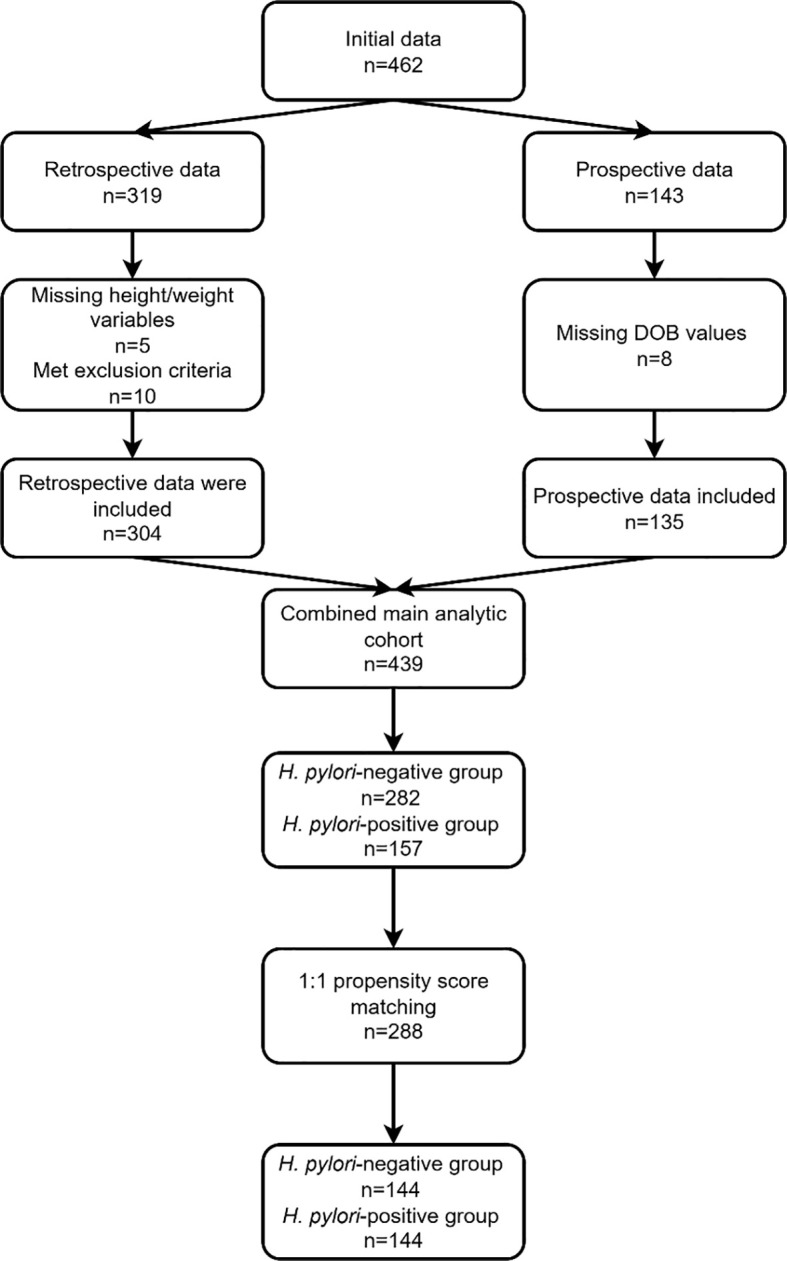
Flowchart of participant selection and propensity score matching.

This was a single-center ambispective observational study. A total of 462 participants who underwent both ¹³C-UBT and colonoscopy during the same diagnostic workup at our hospital were initially enrolled, including 319 retrospective cases and 143 prospective cases. The same inclusion and exclusion criteria were applied to both retrospective and prospective cases. Among the retrospective cases, 5 were excluded because of missing height or weight data, and 10 were excluded according to the exclusion criteria. Among the prospective cases, 8 were excluded because of missing DOB values. Ultimately, 439 participants were included in the main analytic cohort, including 157 *H. pylori*-positive and 282 *H. pylori*-negative individuals. After propensity score matching, 144 matched pairs were obtained for subsequent analyses (**Figure 1**).

Inclusion criteria:

- age ≥18 years;
- complete clinical data;
- Boston Bowel Preparation Scale (BBPS) score ≥6, with a score ≥2 for each colonic segment.

Exclusion criteria:

- previous diagnosis of colorectal cancer or history of partial colorectal resection;
- confirmed inflammatory bowel disease, familial adenomatous polyposis, or other hereditary colorectal tumor syndromes;
- BBPS score <6 or a score <2 in any colonic segment;
- missing key variables or incomplete clinical data.

### 2.2 Study procedures

#### 2.2.1 General clinical data

Basic clinical information was collected, including sex, age, height, weight, smoking, alcohol use, hypertension, and diabetes. Smoking, alcohol use, hypertension, and diabetes were recorded as binary variables.

#### 2.2.2 ¹³C-urea breath test

All enrolled participants underwent standardized ¹³C-UBT. According to the testing protocol at our hospital, participants were asked before the test whether they had recently used antibiotics or proton pump inhibitors (PPIs). If such medication use was reported, the test was not performed. The procedure was as follows: (a) after fasting for at least 2 hours, a baseline breath sample was collected at 0 minutes and securely sealed; (b) the participant ingested a capsule containing 75 mg of ¹³C-urea, with timing initiated immediately thereafter; (c) a second breath sample was collected 30 minutes after ingestion; and (d) both samples were analyzed using an isotope ratio mass spectrometer. According to the test kit instructions and relevant diagnostic criteria, a DOB value ≥4.0‰ was defined as *H. pylori* positive, whereas a DOB value <4.0‰ was defined as *H. pylori* negative. *H. pylori* infection status was entered into the statistical models as a binary variable. In addition to infection status, DOB values were further analyzed as a continuous indicator reflecting urease activity rather than absolute bacterial load.

#### 2.2.3 Colonoscopy

All participants underwent the same bowel preparation regimen until clear watery stool was achieved.

The primary outcome of this study was the presence of colorectal polyps. Outcome classification was based on colonoscopic findings and further confirmed by pathological examination in participants with detected polyps. Pathological results were available for all participants with colorectal polyps. Participants without colorectal polyps on colonoscopy were assigned to the non-polyp group, whereas those with colorectal polyps were assigned to the polyp group. Polyp-related characteristics, including number, maximum diameter, anatomical location, and histological findings, were recorded and descriptively analyzed. Adenomatous polyps were analyzed as a secondary outcome. Adenomatous polyps were defined as conventional adenomas, including tubular adenomas, villous adenomas, and tubulovillous adenomas. In the present cohort, adenomatous polyps included low-grade tubular adenomas, high-grade tubular adenomas, and tubulovillous adenomas according to the available pathological reports. Hyperplastic polyps and serrated lesions were described separately and were not included in the adenomatous polyp outcome.

### 2.3 Statistical analysis

Baseline characteristics before and after propensity score matching were described and compared. Continuous variables were first tested for normality. Normally distributed variables were expressed as mean ± standard deviation, and comparisons between groups were performed using the independent-samples *t* test before matching and the paired *t* test after matching. Non-normally distributed continuous variables were expressed as median (interquartile range), and comparisons were performed using the Mann–Whitney U test before matching and the Wilcoxon signed-rank test after matching. Categorical variables were expressed as counts and percentages. Before matching, comparisons were performed using the chi-square test or Fisher’s exact test; after matching, McNemar’s test or exact McNemar’s test was used for binary variables defined on the basis of the entire matched sample.

To minimize confounding caused by baseline differences, a logistic regression model was constructed with *H. pylori* infection status as the dependent variable and age, sex, BMI, smoking, alcohol use, hypertension, and diabetes as covariates to estimate propensity scores. One-to-one nearest-neighbor matching without replacement was performed using a caliper of 0.02 on the raw propensity score scale. Covariate balance before and after matching was evaluated using standardized differences.

Regression models were constructed to analyze the associations of H. pylori infection status and DOB values with colorectal polyp detection. In the unmatched cohort, univariable logistic regression was first performed, followed by multivariable logistic regression adjusting for age, sex, BMI, smoking, alcohol use, hypertension, and diabetes, with adjusted odds ratios (ORs) and 95% confidence intervals (CIs) calculated. In the propensity score-matched cohort, generalized estimating equation (GEE) models were applied with matched-pair ID as the clustering unit to account for within-pair correlation. Multivariable GEE models were further adjusted for age, sex, BMI, smoking, alcohol use, hypertension, and diabetes because minor residual imbalance remained after matching.

Adenomatous polyps were analyzed as a secondary outcome to assess whether the associations of *H. pylori* infection and higher DOB levels were consistent with the main findings. In the unmatched cohort, additional analyses were performed as follows. First, among *H. pylori*-positive participants, ln (DOB) was used as a continuous variable to evaluate the association between DOB level and colorectal polyp detection. Second, *H. pylori*-positive participants were further stratified according to DOB quartiles within the infected subgroup, and the association between DOB strata and colorectal polyp detection was assessed. Third, in the total cohort, *H. pylori* infection status and ln (DOB + 1) were simultaneously included in the multivariable logistic regression model to evaluate whether DOB provided information beyond binary *H. pylori* infection status. Because some participants had a DOB value of 0, ln(DOB + 1) was used for transformation. *H. pylori* infection status was still defined according to the original DOB cutoff of ≥4.0‰. Because this study included both retrospective and prospective components, sensitivity analyses were further performed according to data source. The associations of *H. pylori* infection status and DOB level with colorectal polyp detection were analyzed separately in the retrospective and prospective cohorts. As an exploratory analysis, ROC curves were constructed in the unmatched cohort using DOB as a continuous predictor and colorectal polyp detection as the outcome. The AUC was calculated, and the optimal cutoff value was determined using the Youden index. Sensitivity and specificity were reported. ROC analyses were also repeated in subgroups defined by smoking, alcohol use, diabetes, and hypertension. Because no external validation was performed, ROC findings were interpreted as exploratory.

All statistical analyses were performed using SPSS version 25.0 and Python version 3.12. Participants with missing key variables, including DOB, height, weight, or other main analytic variables, were excluded before analysis, and complete-case analysis was performed. Because this was an ambispective observational study based on all available eligible participants during the predefined study period, no formal *a priori* sample size calculation was performed. All tests were two-sided, and P<0.05 was considered statistically significant.

This study was approved by the Medical Ethics Committee of our hospital (approval No. 2025-1w012-01). Written informed consent was obtained from participants in the prospective cohort. For retrospective cases, the requirement for informed consent was waived by the ethics committee because de-identified clinical data were used.

## 3 Results

### 3.1 Comparison of baseline characteristics before and after matching

Before propensity score matching, there were 282 participants in the *H. pylori*-negative group and 157 in the *H. pylori*-positive group. Prior to matching, the proportion of men was higher in the *H. pylori*-positive group than in the *H. pylori*-negative group. The proportions of smokers and drinkers were also higher in the *H. pylori*-positive group. BMI was higher in the *H. pylori*-positive group, and the prevalence of diabetes was also higher, whereas no statistically significant differences were observed between the two groups in age or the prevalence of hypertension. Using *H. pylori* infection status as the grouping variable, age, sex, BMI, smoking, alcohol use, hypertension, and diabetes were included in the propensity score model. A total of 144 successfully matched pairs were ultimately obtained, yielding 288 participants, with 144 participants in each of the *H. pylori*-positive and *H. pylori*-negative groups. After matching, baseline differences between the two groups were substantially reduced, and the major covariates were generally balanced. Because minor residual imbalance remained for some covariates, adjusted models were further applied in the matched cohort (**Table 1**).

**Table 1:** Comparison of baseline characteristics between *H. pylori* positive and negative groups before and after propensity score matching.

Variable		Before matching				After matching			
		*H. pylori*-negative group(n=282)	*H. pylori*-positive group (n=157)	SMD	*P*	*H. pylori*-negative group(n=144)	*H. pylori*-positive group(n=144)	SMD	*P*
Sex n (%)	Female	141 (50.00)	53 (33.76)	0.334	0.001	50 (34.72)	53 (36.81)	0.043	0.712
	Male	141 (50.00)	104 (66.24)			94 (65.28)	91 (63.19)		
Age	M(Q_1_, Q_3_)	56.00 (48.00, 61.00)	54.00 (44.00, 60.00)	0.192	0.082	53.00 (45.00, 60.00)	54.00 (44.00, 60.00)	0.068	0.582
BMI	M(Q_1_, Q_3_)	23.03 (21.09, 25.17)	24.22 (22.03, 26.40)	0.313	0.001	23.83 (21.64, 26.40)	24.00 (21.72, 25.86)	0.006	0.946
Smoking n (%)	No	251 (89.01)	126 (80.25)	0.244	0.012	117 (81.25)	119 (82.64)	0.036	0.759
	Yes	31 (10.99)	31 (19.75)			27 (18.75)	25 (17.36)		
Alcohol n (%)	No	265 (93.97)	137 (87.26)	0.232	0.015	130 (90.28)	130 (90.28)	0.000	1
	Yes	17 (6.03)	20 (12.74)			14 (9.72)	14 (9.72)		
Diabetes n (%)	No	253 (89.72)	125 (79.62)	0.283	0.003	125 (86.81)	118 (81.94)	0.134	0.256
	Yes	29 (10.28)	32 (20.38)			19 (13.19)	26 (18.06)		
Hypertension n (%)	No	223 (79.08)	123 (78.34)	0.018	0.857	105 (72.92)	113 (78.47)	0.130	0.272
	Yes	59 (20.92)	34 (21.66)			39 (27.08)	31 (21.53)		

### 3.2 Associations of *H. pylori* status and DOB level with colorectal polyp detection

In the unmatched cohort, 110 colorectal polyps were detected in the *H. pylori*-positive group, corresponding to a detection rate of 70.06%, whereas 94 colorectal polyps were detected in the *H. pylori*-negative group, corresponding to a detection rate of 33.33%; the difference between the two groups was statistically significant. After propensity score matching, 99 colorectal polyps were detected in the *H. pylori*-positive group, corresponding to a detection rate of 68.75%, whereas 76 colorectal polyps were detected in the *H. pylori*-negative group, corresponding to a detection rate of 52.78%; this difference also remained statistically significant (**Table 2A**).

**Table 2A:** Associations of *H. pylori* status and DOB level with colorectal polyp detection before and after propensity score matching. **Table 2(A)**. Detection rate of colorectal polyps according to *H. pylori* status before and after propensity score matching.

*H. pylori* status	Before matching			After matching		
	Total, n	Polyp detected, n (%)	P value	Total, n	Polyp detected, n (%)	P value
Negative	282	94 (33.33)	<0.001	144	76 (52.78)	0.007
Positive	157	110 (70.06)		144	99 (68.75)	

P values were calculated using the Pearson chi-square test before matching and the exact McNemar test after matching.

Logistic regression models were used in the unmatched cohort to evaluate the association between *H. pylori* status and colorectal polyp detection. In the propensity score-matched cohort, generalized estimating equation (GEE) models were applied to account for the correlation within matched pairs. In the unmatched cohort, univariable logistic regression showed that *H. pylori*-positive participants had higher odds of colorectal polyp detection than *H. pylori*-negative participants (OR = 4.68, 95% CI: 3.07–7.14, *P* < 0.001). After adjustment for sex, smoking, alcohol use, diabetes, hypertension, age, and BMI in the multivariable model, the association between *H. pylori* infection status and colorectal polyps remained statistically significant (OR = 4.05, 95% CI: 2.60–6.30, *P* < 0.001) (**Table 2B**).

**Table 2B:** Regression analyses of *H. pylori* status and DOB level with colorectal polyp detection before and after propensity score matching.

Variable	Before matching				After matching			
	Univariable		Multivariable		Univariable		Multivariable	
	OR (95%CI)	*P*	OR (95%CI)	*P*	OR (95%CI)	*P*	OR (95%CI)	*P*
H. pylori status								
Negative	1.00 (Ref)	–	1.00 (Ref)	–	1.00 (Ref)	–	1.00 (Ref)	–
Positive	4.68 (3.07~7.14)	<0.001	4.05 (2.60~6.30)	<0.001	1.97 (1.23~3.14)	0.005	1.91 (1.19~3.07)	0.005
DOB	1.06 (1.04-1.07)	<0.001	1.05 (1.03-1.07)	<0.001	1.03 (1.01-1.05)	0.002	1.03 (1.01~1.05)	<0.001

Logistic regression models were used before matching, and generalized estimating equation models were used after matching to account for within-pair correlation.

In the propensity score-matched sample, GEE models showed that *H. pylori* positivity remained associated with higher odds of colorectal polyp detection compared with *H. pylori* negativity (OR=1.97, 95% CI: 1.23–3.14, *P*=0.005). After further adjustment for sex, smoking, alcohol use, diabetes, hypertension, age, and BMI, this association remained statistically significant (OR=1.91, 95% CI: 1.19–3.07, *P*=0.005) (**Table 2B**). These results suggest that the association between *H. pylori* positivity and colorectal polyp detection persisted after balancing major baseline differences. When DOB was analyzed as a continuous variable, higher DOB levels were associated with increased odds of colorectal polyp detection in the unmatched cohort. This association remained significant after multivariable adjustment (OR = 1.05, 95% CI: 1.03-1.07, P<0.001). Similar results were observed in the propensity score-matched cohort using GEE models, indicating that the association between DOB level and colorectal polyp detection was robust after accounting for matched pairs and residual covariate imbalance (OR = 1.03, 95% CI: 1.01-1.05, P<0.001) (**Table 2B**).

### 3.3 Association of DOB level with colorectal polyp detection among *H. pylori*-positive participants

Considering that DOB-stratified analysis in the total cohort may be influenced by *H. pylori* infection status, we further analyzed the association between DOB level and colorectal polyp detection among *H. pylori*-positive participants. In this subgroup, DOB was natural log-transformed, and ln(DOB) was entered into logistic regression models as a continuous variable. The results showed that higher ln(DOB) remained associated with colorectal polyp detection in the univariable model (OR = 1.66, 95% CI: 1.08–2.57, P = 0.022), and this association remained statistically significant after multivariable adjustment (OR = 2.04, 95% CI: 1.24–3.37, P = 0.005) (**Supplementary Table 1**). Among *H. pylori*-positive participants, DOB values were further divided into quartiles, with approximate cutoffs of 10.33, 20.21, and 35.30‰. As the DOB stratum increased, the detection rate of colorectal polyps showed a progressive upward trend: 55.00% in Q1, 71.79% in Q2, 76.92% in Q3, and 76.92% in Q4. In the multivariable model, higher DOB quartiles were generally associated with higher odds of colorectal polyp detection, and the trend across quartiles remained statistically significant after multivariable adjustment(P for trend=0.004) (**Table 3**).

**Table 3:** Association of DOB level with colorectal polyp detection among *H. pylori*-positive participants.

DOB quartile	Total cases	Cases with colorectal polyps	Detection rate (%)	Univariable OR (95% CI)	P value	Multivariable OR (95% CI)	P value
Q1	40	22	55.00	1.00 (Reference)	–	1.00 (Reference)	–
Q2	39	28	71.79	2.08 (0.82-5.31)	0.124	1.91 (0.65-5.60)	0.238
Q3	39	30	76.92	2.73 (1.03-7.20)	0.043	3.43 (1.10-10.65)	0.033
Q4	39	30	76.92	2.73 (1.03-7.20)	0.043	5.21 (1.55-17.53)	0.008
OR per quartile increase	–	–	–	1.42 (1.03-1.94)	0.031	1.75 (1.20-2.56)	0.004

The analysis included *H. pylori*-positive participants from the unmatched cohort (n = 157). DOB quartiles were defined within *H. pylori*-positive participants. The multivariable model was adjusted for sex, age, smoking, alcohol use, hypertension, diabetes, and BMI. The row labeled OR per quartile increase was used to evaluate the trend across DOB quartiles.

### 3.4 Polyp characteristics and adenomatous polyps as a secondary outcome

In the propensity score-matched cohort, polyp characteristics were further compared among participants with detected colorectal polyps. The proportions of single and multiple polyps were similar between the *H. pylori*-positive and *H. pylori*-negative groups. However, the distribution of maximum polyp size differed between the two groups (P = 0.031); compared with the *H. pylori*-negative group, the *H. pylori*-positive group had a lower proportion of polyps <0.5 cm and relatively higher proportions of polyps measuring 0.5–1.0 cm and >1.0 cm. No significant between-group differences were observed in histological type or anatomical location. Histological findings were mainly hyperplastic polyps and tubular adenomas with low-grade dysplasia, whereas tubular adenomas with high-grade dysplasia, serrated lesions, and tubulovillous adenomas were uncommon. The detailed results are shown in **Supplementary Table 2**.

When adenomatous polyps were analyzed as a secondary outcome, the detection rate of adenomatous polyps was higher in the *H. pylori*-positive group than in the *H. pylori*-negative group (50.00% *vs* 35.42%, P = 0.017). Regression analyses further showed that *H. pylori* positivity and higher DOB levels were associated with increased odds of adenomatous polyp detection. The detailed results are shown in **Supplementary Table 3**.

### 3.5 Sensitivity analyses

In the retrospective cohort, 304 participants were included. Among them, 201 were *H. pylori*-negative, of whom 63 had colorectal polyps, corresponding to a detection rate of 31.34%. Among the 103 *H. pylori*-positive participants, 65 had colorectal polyps, corresponding to a detection rate of 63.11%. The difference in colorectal polyp detection rate between the two groups was statistically significant (P<0.001). In the prospective cohort, 135 participants were included. Among them, 81 were *H. pylori*-negative, of whom 31 had colorectal polyps, corresponding to a detection rate of 38.27%. Among the 54 *H. pylori*-positive participants, 45 had colorectal polyps, corresponding to a detection rate of 83.33%. The difference between the two groups was also statistically significant (**Supplementary Table 4**). Binary logistic regression analyses were further performed using colorectal polyp detection as the dependent variable and *H. pylori* infection status or DOB level as the independent variable. The results showed that *H. pylori* positivity and higher DOB levels were associated with colorectal polyp detection in both the retrospective and prospective cohorts, and these associations remained statistically significant after multivariable adjustment (**Supplementary Table 5**).

In the unmatched total cohort, colorectal polyp detection was used as the outcome, and *H. pylori* infection status and ln(DOB + 1) were simultaneously included in the multivariable logistic regression model. The results showed that ln(DOB + 1) remained associated with colorectal polyp detection, whereas the association for *H. pylori* infection status was attenuated and no longer statistically significant. The detailed results are shown in **Supplementary Table 6**.

### 3.6 Exploratory ROC analysis

In the unmatched cohort, exploratory ROC analysis showed that DOB had limited-to-moderate discriminatory ability for colorectal polyp detection, with an AUC of 0.713, an optimal cutoff value of 4.57, a sensitivity of 53.43%, and a specificity of 82.55%. Subgroup ROC analyses stratified by smoking, alcohol consumption, diabetes, and hypertension are presented in **Supplementary Figure 1** and **Supplementary Table 7**. Given the observational design, lack of external validation, and limited sensitivity of DOB as a single marker, these findings should be interpreted as exploratory.

## 4 Discussion

This study analyzed the associations of *H. pylori* infection status and DOB values with colorectal polyp detection among adults who underwent both ¹³C-UBT and total colonoscopy during the same diagnostic workup at our hospital between 2023 and 2024. The results showed that, in both the unmatched cohort and the propensity score-matched sample, the detection rate of colorectal polyps was higher in the *H. pylori*-positive group than in the *H. pylori*-negative group. After adjustment for age, sex, BMI, smoking, alcohol use, hypertension, and diabetes, this association remained statistically significant. In addition, higher DOB values were also associated with colorectal polyp detection. Further analyses among *H. pylori*-positive participants showed that ln(DOB) and DOB quartiles were associated with colorectal polyp detection, suggesting that the association with DOB was not completely explained by the binary definition of *H. pylori* positivity. In the matched cohort, the two groups showed no significant differences in polyp number, histological category, or anatomical location, whereas the distribution of maximum polyp size was different. When adenomatous polyps were analyzed as a secondary outcome, the detection rate was higher in the *H. pylori*-positive group, but this result should be interpreted cautiously because some histological subtypes were uncommon.

Our findings are generally consistent with most observational studies supporting an association between *H. pylori* and colorectal lesions (8, 9, 12, 13). Previous systematic reviews and meta-analyses have shown that *H. pylori* positivity is associated with a higher likelihood of benign colorectal polyps, colorectal adenomas, and advanced adenomas, and that this association tends to be more pronounced in Asian populations (8). At the same time, some studies have failed to find this positive association (10, 11, 14, 15). Possible reasons for these inconsistencies include differences in study populations, variation in the measurement of *H. pylori* exposure, timing between *H. pylori* assessment and colonoscopy, and inconsistent definitions of study outcomes (8, 10). In the present study, *H. pylori* status was assessed using ¹³C-UBT, and the breath test and colonoscopy were performed during the same diagnostic workup, which may have reduced exposure misclassification and temporal bias. Therefore, our findings may be closer to those reported in studies using markers of active infection.

In addition to *H. pylori* infection status, we further analyzed DOB as a continuous UBT-derived indicator. The results showed that higher DOB values were associated with higher odds of colorectal polyp detection in both the unmatched cohort and the matched sample. Considering that DOB stratification in the total cohort may partly reflect the diagnostic threshold for *H. pylori* positivity, we further performed analyses restricted to *H. pylori*-positive participants. In this subgroup, ln (DOB) and higher DOB quartiles were also associated with colorectal polyp detection. These findings suggest that the association between *H*. pylori infection and colorectal polyps may not be limited to the binary distinction of infection status, but may also be related to differences in DOB values among infected individuals.

Compared with traditional binary models, the use of continuous exposure and stratified analyses may provide a more detailed assessment of the graded association between DOB levels and colorectal polyp detection. However, given the observational design and the uncertainty regarding the biological interpretation of DOB, these findings should be interpreted cautiously.

Exploratory ROC analysis in this study showed that the AUC of DOB for discriminating the presence of colorectal polyps was 0.713 in the unmatched cohort, indicating that its discriminatory ability as a single marker was limited to moderate. At the optimal cutoff value of 4.57, the sensitivity was 53.43% and the specificity was 82.55%. Further subgroup analyses according to smoking, alcohol use, diabetes, and hypertension status were also performed. However, because some subgroup sample sizes were small and no external validation was performed, these findings should still be interpreted primarily as exploratory. Although DOB cannot be used as a standalone screening marker, it may provide supplementary information when combined with established clinical factors, but it should not replace standard colorectal cancer screening or colonoscopy-based evaluation.

This study has several limitations. First, as a single-center observational study based on a regional population, it has limited capacity for causal inference and restricted generalizability. In addition, the study population consisted of hospital-based participants who underwent both ¹³C-UBT and colonoscopy during the same diagnostic workup, and selection bias may therefore have existed. Second, although major confounding factors were adjusted for as much as possible, residual confounding cannot be completely excluded. Information on dietary pattern, physical activity, red meat intake, family history of colorectal cancer, previous history of colorectal polyps, aspirin or NSAID use, PPI use, antibiotics or bismuth exposure, previous *H. pylori* eradication therapy, colonoscopy indication, endoscopist-related factors, and withdrawal time was not fully available, and these factors may have influenced the results. Third, because *H. pylori* assessment and colorectal polyp detection were performed during the same diagnostic workup, the temporal sequence between the two could not be determined. Therefore, the present study can only suggest associations rather than causality. Fourth, although pathological results were available for all participants with detected colorectal polyps, some histological subtypes, including high-grade adenomas, serrated lesions, and tubulovillous adenomas, were uncommon, which limited further subtype-specific analyses. In addition, DOB values mainly reflect the results of ¹³C-UBT under a specific testing system and may be influenced by gastric environment, gastric emptying, atrophic gastritis, medication exposure, previous eradication therapy, and testing platform. Therefore, DOB values are not equivalent to absolute bacterial load. The DOB-related trend observed in this study should be interpreted as a statistical finding based on this specific sample and testing system, rather than being directly generalized as a definite biological dose-response relationship or a universally applicable clinical threshold. Finally, the ROC analysis in this study was exploratory and lacked external validation. The discriminatory value of DOB as a single marker still needs to be further evaluated in larger multicenter studies.

## 5 Conclusion

In this single-center ambispective observational study, *H. pylori* positivity and higher UBT-derived DOB values were associated with increased odds of colorectal polyp detection. Similar DOB-related associations were observed among *H. pylori*-positive participants, and adenomatous polyps were detected at a higher rate in the *H. pylori*-positive group. However, DOB alone showed limited discriminatory ability. Further multicenter studies with more comprehensive confounding control and pathological classification are needed to validate these findings.

## Data Availability

The raw data supporting the conclusions of this article will be made available by the authors, without undue reservation.
