# Supplementary material for: Association between H. pylori infection status, UBT-derived DOB level, and colorectal polyp detection
Source: Front Oncol. 2026 Jun 9;16:1851795. doi: 10.3389/fonc.2026.1851795 (PMC13286796; doi:10.3389/fonc.2026.1851795)
Supplement: Supplementary file 1 [file DataSheet1.docx]

**Supplementary Table S1. Logistic regression analysis of ln(DOB) and colorectal polyp detection among H. pylori-positive participants**

| **Variable** | **Model** | **OR (95% CI)** | **P value** |
| --- | --- | --- | --- |
| ln(DOB) | Univariable model | 1.66 (1.08-2.57) | 0.022 |
| ln(DOB) | Multivariable model | 2.04 (1.24-3.37) | 0.005 |

Note: The analysis included H. pylori-positive participants from the unmatched cohort (n = 157). The outcome was the presence of colorectal polyps. The multivariable model was adjusted for sex, age, smoking, alcohol use, hypertension, diabetes, and BMI. DOB, delta over baseline; OR, odds ratio; CI, confidence interval.

**Supplementary Table S2. Characteristics of colorectal polyps according to H. pylori status after propensity score matching**

| **Characteristics** | **H. pylori-negative (n=76)** | **H. pylori-positive (n=99)** | **P value** |
| --- | --- | --- | --- |
| **Polyp number** |  |  | 0.755 |
| Single | 45 (59.21) | 62 (62.63) |  |
| Multiple | 31 (40.79) | 37 (37.37) |  |
| **Maximum polyp size** |  |  | 0.031 |
| <0.5 cm | 33 (43.42) | 26 (26.26) |  |
| 0.5-1.0 cm | 41 (53.95) | 65 (65.66) |  |
| >1.0 cm | 2 (2.63) | 8 (8.08) |  |
| **Histological type** |  |  | 0.240 |
| Hyperplastic polyps | 25 (32.89) | 25 (25.25) |  |
| Low-grade tubular adenoma | 51 (67.11) | 68 (68.69) |  |
| High-grade tubular adenoma | 0 (0.00) | 2 (2.02) |  |
| Serrated lesions | 0 (0.00) | 2 (2.02) |  |
| Tubulovillous adenomas | 0 (0.00) | 2 (2.02) |  |
| **Location** |  |  |  |
| Rectum | 18 (23.68) | 24 (24.24) | 1.000 |
| Sigmoid colon | 19 (25.00) | 33 (33.33) | 0.248 |
| Descending colon | 14 (18.42) | 32 (32.32) | 0.056 |
| Transverse colon | 23 (30.26) | 28 (28.28) | 0.867 |
| Ascending colon | 24 (31.58) | 30 (30.30) | 0.870 |

Note: Data are presented as n (%). Serrated lesions were described separately and were not included in the adenomatous polyp outcome. For anatomical location, categories were not mutually exclusive because some participants had polyps in more than one colonic segment.

**Supplementary Table S3. Adenomatous polyps as a secondary outcome after propensity score matching**

**A. Detection rate of adenomatous polyps according to H. pylori status**

| **H. pylori status** | **Total participants** | **Adenomatous polyps** | **Detection rate (%)** | **P value** |
| --- | --- | --- | --- | --- |
| Negative | 144 | 51 | 35.42 | 0.017 |
| Positive | 144 | 72 | 50.00 |  |

Note: P value was calculated using the exact McNemar test.

**B. Generalized estimating equation analyses using adenomatous polyps as the outcome**

| **Exposure** | **Univariable OR (95% CI)** | **P value** | **Multivariable OR (95% CI)** | **P value** |
| --- | --- | --- | --- | --- |
| H. pylori-positive vs negative | 1.82 (1.14-2.93) | 0.013 | 1.80 (1.09-2.97) | 0.022 |
| DOB, per 1‰ increase | 1.02 (1.00-1.03) | 0.012 | 1.02 (1.01-1.04) | 0.005 |

Note: Adenomatous polyps included low-grade tubular adenomas, high-grade tubular adenomas, and tubulovillous adenomas. Multivariable models were adjusted for sex, age, smoking, alcohol use, hypertension, diabetes, and BMI.

**Supplementary Table S4. Detection rate of colorectal polyps according to *H. pylori* status stratified by data source**

| **Data source** | ***H. pylori* status** | **Total cases** | **Cases with colorectal polyps** | **Cases without colorectal polyps** | **Detection rate (%)** | **Statistic** | **P value** |
| --- | --- | --- | --- | --- | --- | --- | --- |
| Retrospective cohort | Negative | 201 | 63 | 138 | 31.34 | chi-square = 28.187 | <0.001 |
|  | Positive | 103 | 65 | 38 | 63.11 |  |  |
| Prospective cohort | Negative | 81 | 31 | 50 | 38.27 | chi-square = 26.740 | <0.001 |
|  | Positive | 54 | 45 | 9 | 83.33 |  |  |

**Supplementary Table S5. Associations of *H. pylori* status and DOB with colorectal polyp detection stratified by data source**

| **Exposure** | **Data source** | **Univariable OR (95% CI)** | **P value** | **Multivariable OR (95% CI)** | **P value** |
| --- | --- | --- | --- | --- | --- |
| *H. pylori* positive vs negative | Retrospective cohort | 3.75 (2.27-6.17) | <0.001 | 3.45 (2.05-5.79) | <0.001 |
| *H. pylori* positive vs negative | Prospective cohort | 8.06 (3.47-18.76) | <0.001 | 7.05 (2.80-17.77) | <0.001 |
| DOB, per 1‰ increase | Retrospective cohort | 1.06 (1.03-1.08) | <0.001 | 1.05 (1.03-1.08) | <0.001 |
| DOB, per 1‰ increase | Prospective cohort | 1.06 (1.02-1.09) | 0.001 | 1.05 (1.02-1.09) | 0.002 |

**Note:** The outcome variable was the presence of colorectal polyps. The multivariable models were adjusted for sex, age, smoking, alcohol consumption, hypertension, diabetes, and BMI.

**Supplementary Table S6. Multivariable logistic regression model including both H. pylori status and ln(DOB+1) in the total cohort**

| **Variable** | **Adjusted OR** | **95% CI** | **P value** |
| --- | --- | --- | --- |
| H. pylori positive vs negative | 0.93 | 0.38-2.25 | 0.872 |
| ln(DOB+1) | 1.96 | 1.37-2.80 | <0.001 |

**Note:** The model included H. pylori status and ln(DOB+1) simultaneously and was adjusted for sex, age, smoking, alcohol use, hypertension, diabetes, and BMI. ln(DOB+1) was used only as a transformed continuous variable to account for skewness and zero values.

Supplementary Figure S1. Exploratory ROC curve of DOB for colorectal polyp detection in the unmatched cohort.


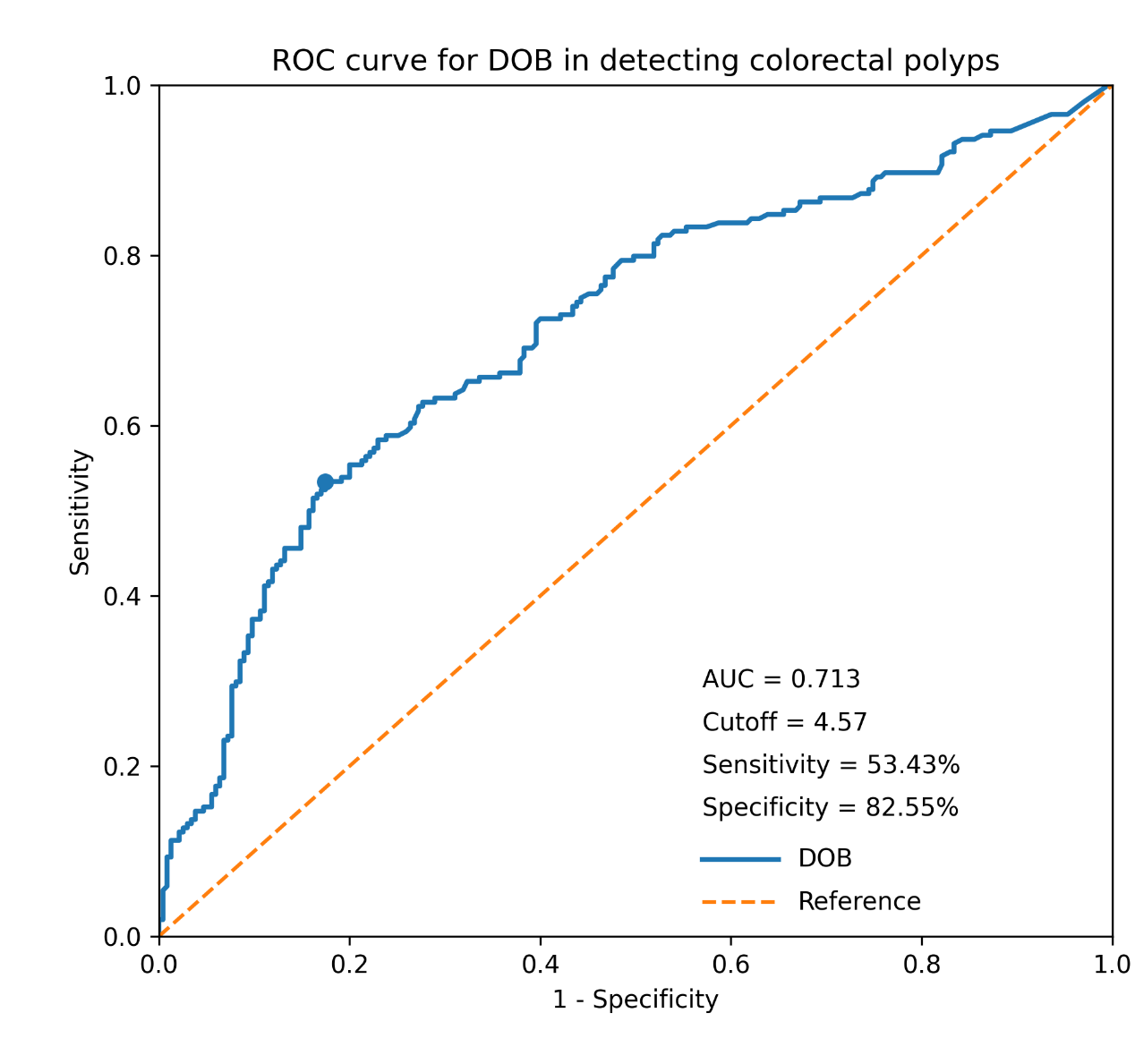


ROC curve of DOB for colorectal polyp detection in the unmatched cohort. The optimal cutoff was determined by the Youden index. AUC, sensitivity, and specificity are shown in the figure.

**Supplementary Table S7. Exploratory ROC analysis of DOB for colorectal polyp detection in the unmatched cohort and clinical subgroups**

| **Stratification variable** | **Subgroup** | **n** | **Polyp cases** | **AUC (95% CI)** | **Optimal cutoff** | **Sensitivity (%)** | **Specificity (%)** |
| --- | --- | --- | --- | --- | --- | --- | --- |
| Overall | Overall | 439 | 204 | 0.713 (0.661-0.759) | 4.57 | 53.43 | 82.55 |
| Smoking | No | 377 | 162 | 0.701 (0.647-0.755) | 4.57 | 50.62 | 82.79 |
| Smoking | Yes | 62 | 42 | 0.766 (0.639-0.885) | 2.65 | 73.81 | 80.00 |
| Alcohol consumption | No | 402 | 178 | 0.699 (0.644-0.753) | 2.88 | 61.24 | 71.88 |
| Alcohol consumption | Yes | 37 | 26 | 0.808 (0.619-0.952) | 4.91 | 73.08 | 90.91 |
| Diabetes | No | 378 | 163 | 0.709 (0.653-0.759) | 3.31 | 56.44 | 77.21 |
| Diabetes | Yes | 61 | 41 | 0.736 (0.605-0.853) | 4.87 | 65.85 | 80.00 |
| Hypertension | No | 346 | 162 | 0.720 (0.666-0.771) | 4.57 | 53.70 | 83.70 |
| Hypertension | Yes | 93 | 42 | 0.676 (0.562-0.788) | 3.10 | 61.90 | 70.59 |

Notes: The optimal cutoff value was determined using the Youden index. AUC 95% CIs were estimated using 2,000 bootstrap resamples.
